# Cooperative and Independent Functionality of tmRNA and SmpB in *Aeromonas veronii*: A Multifunctional Exploration Beyond Ribosome Rescue

**DOI:** 10.3390/ijms26010409

**Published:** 2025-01-06

**Authors:** Taipeng Bai, Juanjuan Li, Xue Chi, Hong Li, Yanqiong Tang, Zhu Liu, Xiang Ma

**Affiliations:** 1 Pathogenesis and Control of Pathogenic Microorganisms Research Team, School of Life and Health Sciences, Hainan Province Key Laboratory of One Health, Collaborative Innovation Center of One Health, Hainan University, Haikou 570228, China; taipengb@163.com (T.B.); lijuanjuan133@126.com (J.L.); chixue0314@163.com (X.C.); lihongbio@163.com (H.L.); tyq68@126.com (Y.T.); 2 Yunnan Provincial Key Laboratory of Animal Nutrition and Feed, Faculty of Animal Science and Technology, Yunnan Agricultural University, Kunming 650201, China; zhuliu@ynau.edu.cn

**Keywords:** tmRNA, SmpB, trans-translation, transcriptomic, stress response, siderophore

## Abstract

The trans-translation system, mediated by transfer-messenger RNA (tmRNA, encoded by the *ssrA* gene) and its partner protein SmpB, helps to release ribosomes stalled on defective mRNA and targets incomplete protein products for hydrolysis. Knocking out the *ssrA* and *smpB* genes in various pathogens leads to different phenotypic changes, indicating that they have both cooperative and independent functionalities. This study aimed to clarify the functional relationships between tmRNA and SmpB in *Aeromonas veronii,* a pathogen that poses threats in aquaculture and human health. We characterized the expression dynamics of the *ssrA* and *smpB* genes at different growth stages of the pathogen, assessed the responses of deletion strains Δ*ssrA* and Δ*smpB* to various environmental stressors and carbon source supplementations, and identified the gene-regulatory networks involving both genes by integrating transcriptomic and phenotypic analyses. Our results showed that the gene *ssrA* maintained stable expression throughout the bacterial growth period, while *smpB* exhibited upregulated expression in response to nutrient deficiencies. Compared to the wild type, both the Δ*ssrA* and Δ*smpB* strains exhibited attenuated resistance to most stress conditions. However, Δ*ssrA* independently responded to starvation, while Δ*smpB* specifically showed reduced resistance to lower concentrations of Fe^3+^ and higher concentrations of Na^+^ ions, as well as increased utilization of the carbon source β-Methyl-D-glucoside. The transcriptomic analysis supported these phenotypic results, demonstrating that tmRNA and SmpB cooperate under nutrient-deficient conditions but operate independently in nutrient-rich environments. Phenotypic experiments confirmed that SsrA and SmpB collaboratively regulate genes involved in siderophore synthesis and iron uptake systems in response to extracellular iron deficiency. The findings of the present study provide crucial insights into the functions of the trans-translation system and highlight new roles for tmRNA and SmpB beyond trans-translation.

## 1. Introduction

Translation is a crucial biological process of protein synthesis within organisms, tightly regulated to ensure efficiency and accuracy [1]. During translation, if a truncated mRNA is encountered, the ribosome stalls on it, depleting the available ribosome pool. Furthermore, truncated mRNA may encode abnormally toxic proteins, posing a significant threat to the organism’s survival [2]. The mRNA surveillance pathway is essential for accurate gene expression and maintaining translational homeostasis, with trans-translation serving as the primary quality control system for protein synthesis that has evolved in bacteria [3]. Transfer-messenger RNA (tmRNA), which is the core component of the trans-translation system, consists of a tRNA-like domain that carries alanine and an mRNA-like domain that encodes the SsrA degradation tag and stop codon. tmRNA is delivered to the ribosome’s A site by its partner proteins SmpB and EF-Tu-GTP, enabling the stalled ribosome to switch templates and resume the translation of the coding sequence within the tmRNA molecule [4].

Trans-translation is conserved across bacteria and is essential in many species [5]. Previous studies have demonstrated that the SmpB-SsrA system plays a significant biological role. For instance, the *smpB* mutant of *Salmonella typhimurium* exhibits defects in survival within macrophages, while the *ssrA* mutant shows reduced virulence [6,7]. In *Escherichia coli*, both *ssrA* and *smpB* mutants display increased sensitivity to various antibiotics, as well as to stresses such as acids, weak acids, salicylic acid, high temperatures, and peroxides, with *smpB* mutants being more susceptible than *ssrA* mutants [8]. Although numerous studies indicate that the phenotypes resulting from the deletions of the *smpB* gene and the *ssrA* gene are similar [9,10], the mechanisms underlying the increased vulnerability of *smpB* mutants under most stress conditions remain unclear.

Gene expression is influenced not only by the transcription rates but also significantly by post-transcriptional regulation, where the translation rates and mRNA decay play crucial roles in determining the final protein levels [11]. This suggests that the SmpB-SsrA system may be involved in the regulation of gene expression. Research has shown that *ssrA* plays an important role in the transcriptional regulation of *Bacillus subtilis* spores, as the absence of *ssrA* prevents the synthesis of active σ*^k^* [12]. Additionally, *ssrA* can regulate the levels of active Lac repressor and control the cell cycle in *Caulobacter crescentus* by mediating the removal of the regulatory factor CtrA [13,14]. In *Yersinia pseudotuberculosis*, *smpB-ssrA* mutants exhibit severe defects in the expression and secretion of virulence effector proteins, with these defects occurring at the transcriptional level [15]. Furthermore, tmRNA has been shown to act as an antisense RNA targeting the crtMN mRNA, inhibiting the synthesis of pigments in *Staphylococcus aureus* [16]. Studies have also found that SmpB directly or indirectly regulates the expression of at least 4% of the proteome of *S. typhimurium* [17]. These findings indicate that SmpB and tmRNA may play important roles in gene regulation.

Previously, it was believed that tmRNA and SmpB could not function in the absence of each other [18]. However, in *Mycobacterium tuberculosis*, bacteria can survive normally when *smpB* is knocked out, without altering *ssrA* [19]. Additionally, six bacterial species were found to lack only tmRNA, while sixteen species lacked only *smpB*, suggesting that there are more independent functions between tmRNA and SmpB than previously recognized [20]. While most studies focus on revealing the cooperative functions of the two, there has been relatively little attention paid to the pathways and activities in which they participate independently.

*Aeromonas veronii* is found in various aquatic environments and serves as a pathogen for aquatic animals, capable of causing diseases such as skin ulcers and systemic hemorrhagic septicemia in fish [21,22]. Additionally, it is an emerging human intestinal pathogen, frequently identified in patients with inflammatory bowel disease [23]. More than 300 virulence factors have been characterized in *A. veronii*, with aerolysin, microbial collagenase, and various hemolysins detected in all strains isolated from patients suffering from gastrointestinal diseases [24]. Our previous research demonstrated that the knockout of *ssrA* and *smpB* reduces the virulence of *A. veronii* [25], while *smpB* influences its antibiotic resistance [26]. Furthermore, the knockout of *tmRNA* affects its metabolism and resistance to antibiotics that target the cell wall [27].

In this study, we characterized the expression dynamics of *ssrA* and *smpB* in *A. veronii.* We also investigated the changes in the responses of *ssrA* and *smpB* single knockout strains to environmental stress and their ability to utilize various carbon sources. Through transcriptomic analysis, we identified differentially expressed genes to explore the similarities and differences in the regulatory effects of SsrA and SmpB on gene expression. Our findings indicate that SsrA and SmpB collaboratively regulate the utilization of L-asparagine and D-mannitol, as well as the synthesis of iron transporters. However, they exhibit independent or opposing effects in response to starvation, low iron, high sodium stress, peptidoglycan synthesis, and bacterial chemotaxis. Furthermore, we discovered that the cooperation between SsrA and SmpB is enhanced under nutrient-deficient conditions, while SsrA and SmpB demonstrate greater independence in nutrient-rich environments. This study provides significant evidence and critical insights into the novel functions of tmRNA and SmpB in *A. veronii*, which are independent of trans-translation.

## 2. Results

### 2.1. tmRNA Is Stable During Bacterial Growth, Whereas Higher smpB Expression Is a Response to Nutrient Deficiencies

**Figure 1:**
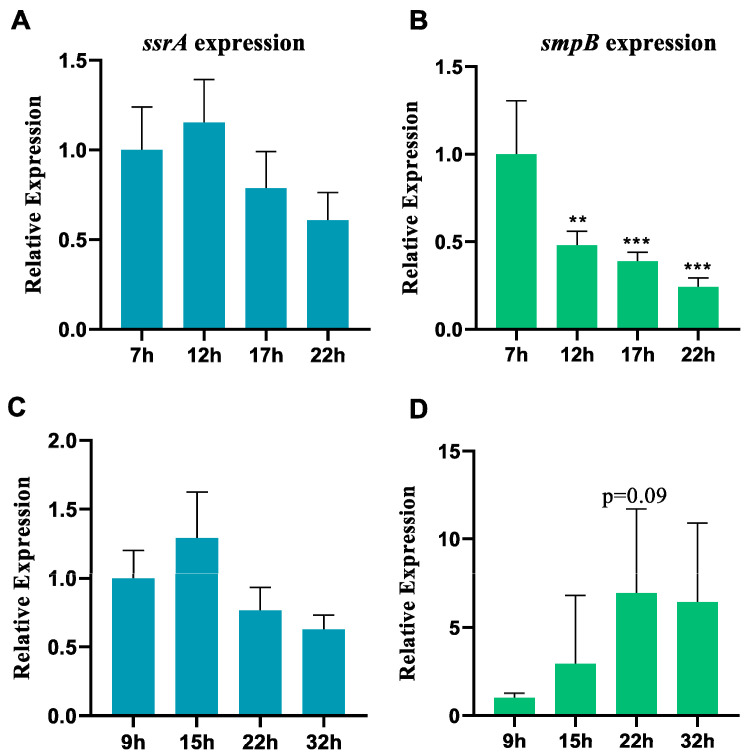
The genes *ssrA* and *smpB* exhibit different expression patterns in response to nutrient deficiency. RT-qPCR was used to determine the relative expression levels of *ssrA* (**A**,**C**) or *smpB* (**B**,**D**) at different times under LB conditions (**A**,**B**) or M9 conditions (**C**,**D**). Tukey’s post-test was used for statistical analysis, with ** representing *p* < 0.01 and *** representing *p* < 0.005 in one-way ANOVA.

Considering that both tmRNA and SmpB are core functional components of the trans-translation system, we examined the expression dynamics of the *ssrA* and *smpB* genes in the wild-type *A. veronii* under nutrient-rich LB and nutrient-deficient M9 culture conditions at different stages of bacterial growth. The results indicate that, under nutrient-rich LB conditions, the expression of the *ssrA* gene remains stable during both the exponential and stationary phases, showing no significant differences during different growth phases (Figure 1A). In contrast, the expression of the *smpB* gene significantly decreases during the transition from the logarithmic to the stationary phase, with the expression levels in the stationary phase (22 h) being approximately 30% of those in the exponential phase (7 h) (Figure 1B). Under nutrient-deficient M9 conditions, the expression of the *ssrA* gene also remains stable during both the exponential and stationary phases, with no significant differences observed (Figure 1C). Conversely, the expression of the *smpB* gene in the stationary phase (22 h) increases by about seven times as compared with that observed in the exponential phase (9 h) (Figure 1D), although the *p*-value is slightly higher than 0.05. Taken together, during the transition from the exponential to the stationary phase, the expression of tmRNA remains stable, while the *smpB* expression is influenced by the nutrient availability. Specifically, the nutrient-rich condition induces the significantly lower *smpB* expression in the stationary phase, whereas the nutrient-deficient condition stimulates a significant increase in *smpB* expression in the stationary phase. This may be due to fewer mRNA translation errors in nutrient-rich environments, leading to the reduced expression of the trans-translation system, while a nutrient deficiency increases the error rates and elevates its expression. This suggests that pathogenic bacteria may regulate the activity and function of the trans-translation system in response to the environment, primarily by adjusting *smpB* expression.

### 2.2. tmRNA and SmpB Collaborate or Independently Participate in Specific Types of Stress Responses

If tmRNA and SmpB take actions in an independent way in the environmental adaptation of pathogenic bacteria, the Δ*ssrA* and Δ*smpB* strains, which are individually deficient in these components, should exhibit distinct phenotypes under the same stress conditions. Therefore, we comprehensively assessed the growth status of the wild-type, Δ*ssrA*, and Δ*smpB* strains under various environmental stress treatments, including iron deficiency, hypoxia, high salinity, and varying pH levels, to explore the potential regulatory pathways in which both components may be involved.

**Figure 2:**
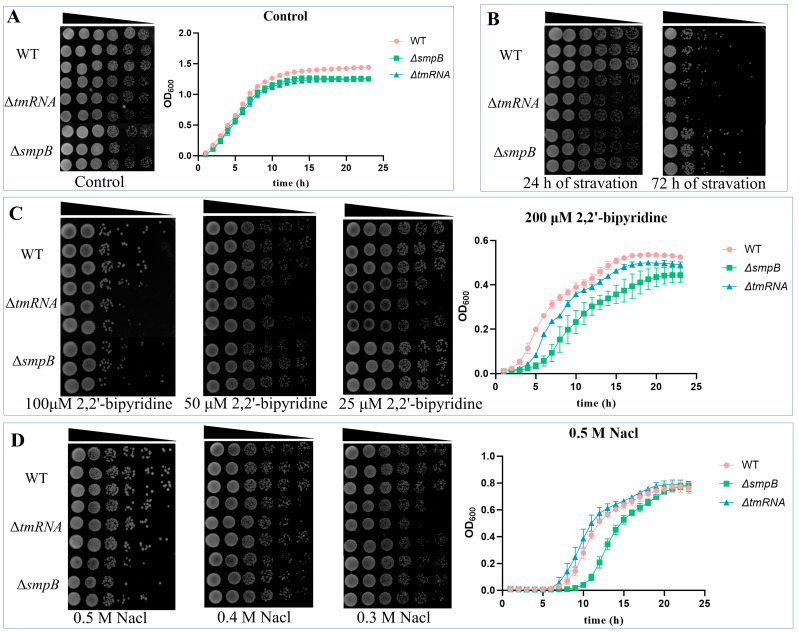
tmRNA and SmpB cooperate or independently participate in the responses to starvation, osmotic pressure, and low iron stress. For the determination of the growth curve, the bacteria were transferred to standard LB medium (**A**, right panel) or LB medium supplemented with 200 μM 2,2′-bipyridine (**C**, right panel) or 0.5 M sodium chloride (**D**, right panel). Data are presented as the mean ± SD from three replicates. For the plate experiment, after the overnight culture was washed with PBS, a ten-fold serial dilution of the bacterial suspension was prepared, and 3 μL of each dilution was spotted onto LB agar plates supplemented with different concentrations of 2,2’-bipyridine (**C**, left panel) or sodium chloride (**D**, left panel). For the starvation treatments, bacterial suspensions were allowed to stand in PBS buffer and dotted on LB plates after 24 h or 72 h (**B**).

Under standard LB culture conditions, the Δ*tmRNA* and Δ*smpB* strains exhibit subtle growth retardances as compared with the wild type (Figure 2A). In contrast, the growth of the Δ*tmRNA* and Δ*smpB* strains demonstrated no significant differences from the wild-type strain after being cultured under starvation conditions for 24 h. However, after 72 h of starvation, the Δ*smpB* strain exhibited growth comparable to that of the wild type, while the Δ*tmRNA* strain displayed a significant decline in growth capacity (Figure 2B). This suggests that tmRNA may independently regulate the starvation tolerance of the strain. A similar phenomenon has been observed in *Escherichia coli* previously [8]. When the environmental concentration of iron ions is reduced by 2,2′-bipyridine chelators, the growth of the Δ*tmRNA* and Δ*smpB* strains shows no significant differences from the wild-type strain at low concentrations. However, at 100 μM 2,2′-bipyridine, both knockout strains exhibit a significant decrease in tolerance, with the Δ*smpB* strain showing a more pronounced decline at 200 μM (Figure 2C). Similarly, treatment with 1 mM EDTA reduces the tolerance in both strains compared to the wild type, with the Δ*smpB* strain demonstrating a more significant reduction (Figure S1A), indicating that the absence of *smpB* specifically impairs the pathogen’s iron acquisition capability. Under salt stress with varying NaCl concentrations, the Δ*tmRNA* and Δ*smpB* strains show no significant differences from the wild type under lower salt conditions. However, at 0.5 M NaCl, the Δ*smpB* strain exhibits a significant decrease in tolerance, while the Δ*tmRNA* strain’s tolerance is nearly identical to that of the wild type. The growth curve analysis is consistent with the results of the plate assay (Figure 2D).

Treatment with bile salts, nitrate, and the metal oxidant CuCl_2_ did not result in significant differences in growth between the Δ*tmRNA* and Δ*smpB* strains compared to the wild-type strain (Figure S1B–D). Moreover, treatments with 1% butanol and 3% ethanol weakened the growth of both the Δ*tmRNA* and Δ*smpB* strains relative to the wild type, with their growth abilities being comparable (Figure S1E,F). This indicates that tmRNA and SmpB do not participate in the stress responses to bile salts, nitrate, and metal oxidants, but they do collaborate in response to alcohol stress. The bacterial growth status under different pH conditions shows that both the Δ*tmRNA* and Δ*smpB* strains exhibit reduced growth compared to the wild type under alkaline conditions, with their growth abilities being similar. However, under neutral and acidic conditions, there are no significant differences in tolerance between the Δ*tmRNA* and Δ*smpB* strains and the wild-type strain (Figure S1G).

Based on these results, it is evident that the individual knockout of the *ssrA* or *smpB* gene significantly reduces the tolerance of *A. veronii* under conditions of low iron, high salinity, starvation, alcohol stress, and alkalinity. The *smpB* gene plays a crucial role when the bacteria face iron deficiency and high osmotic pressure stress, while the *ssrA* gene independently regulates the growth capacity of the pathogen under starvation conditions.

### 2.3. tmRNA and SmpB Collaborate or Independently Participate in the Utilization of Different Types of Carbon Sources

**Figure 3:**
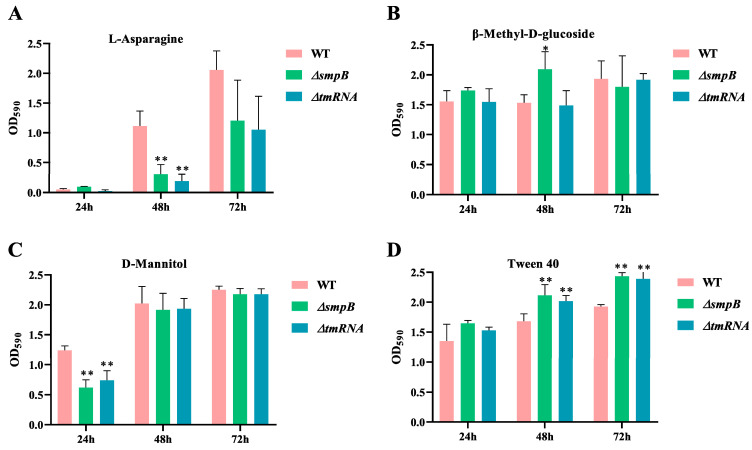
tmRNA and SmpB participate in the metabolism of different types of carbon sources cooperatively or independently. WT, Δ*tmRNA*, and Δ*smpB* were inoculated on a BIOLOG ECO microplate at 30 °C with L-aspartate (**A**), β-Methyl-D-glucoside (**B**), D-mannitol (**C**), and Tween 40 (**D**) as the sole carbon sources. The absorption values were recorded at 590 nm at an interval of 24 h. Data are presented as the mean ± SD from three replicates. Tukey’s post-test was used for statistical analysis, with * representing *p* < 0.05 and ** representing *p* < 0.01 in one-way ANOVA.

Previous studies have demonstrated that the SsrA-SmpB system is involved in purine and amino acid metabolism [28,29]. Our earlier research also indicated that the knockout of the *ssrA* gene affected the accumulation of specific metabolites in *A. veronii* [27]. To further investigate the role of trans-translation in nutrient utilization, we employed the BIOLOG ECO microplate assay to assess the growth capabilities of the wild-type, Δ*tmRNA*, and Δ*smpB* strains of *A. veronii* on 31 different sole carbon sources. The results showed that the individual knockout of either the *ssrA* or *smpB* gene significantly impacted the pathogen’s ability to utilize the carbon sources β-Methyl-D-glucoside, L-asparagine, Tween 40, and D-mannitol compared to the wild-type strain (Figure 3). After 24 h of cultivation, the OD_590_ values of the Δ*tmRNA* and Δ*smpB* strains grown on D-mannitol as the sole carbon source were half that of the wild type. However, by 48 h, the growth levels of all three strains reached parity, indicating that the individual knockout of the *ssrA* or *smpB* genes reduced the utilization rate of D-mannitol by *A. veronii* (Figure 3C). After 48 h, the OD_590_ values for the Δ*tmRNA* and Δ*smpB* strains grown on L-asparagine as the sole carbon source were only 17% and 27% of that of the wild type, respectively; however, this difference diminished over time, suggesting that the individual knockouts also slowed the utilization rate of L-asparagine (Figure 3A). Additionally, the OD_590_ values for the Δ*tmRNA* and Δ*smpB* strains on Tween 40 as the carbon source were 1.2 times that of the wild type, and this growth advantage persisted for up to 72 h (Figure 3D), indicating that the individual knockouts of the *ssrA* or *smpB* genes consistently enhanced the utilization of Tween 40 by *A. veronii*.

These results suggest that the *ssrA* and *smpB* genes work together in the carbon metabolism of *A. veronii* for D-mannitol, L-asparagine, and Tween 40. Surprisingly, the OD value for the Δ*smpB* strain grown on β-Methyl-D-glucoside was 1.3 times that of the wild type, while the growth of the Δ*tmRNA* strain on the same carbon source showed no significant difference from the wild type (Figure 3B). This finding suggests that SmpB may independently regulate the metabolic activity of *A. veronii* towards β-Methyl-D-glucoside.

### 2.4. tmRNA and SmpB Respond to Different Nutritional Conditions and Regulate the Expression of Specific Genes

Our findings indicate that tmRNA and SmpB exhibit collaboration and functional specialization in pathogenic bacteria’s responses to environmental stress and carbon source utilization. To further investigate the mechanisms underlying the consistent or independent phenotypic differences observed in the Δ*ssrA* and Δ*smpB* strains, we performed RNA sequencing on stationary-phase bacteria cultured under nutrient-deficient M9 and nutrient-rich LB conditions. This approach enabled us to screen and analyze the differential gene expression profiles of the Δ*ssrA* and Δ*smpB* strains compared to the wild type.

**Figure 4:**
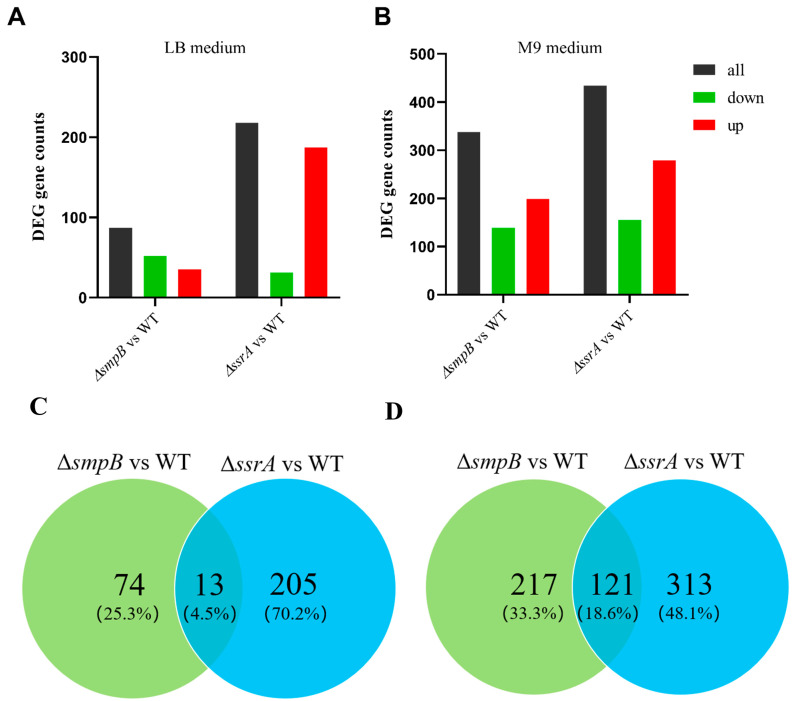
tmRNA and SmpB exhibit enhanced collaboration under nutrient deficiency conditions, but show significant independence in nutrient enrichment conditions. Total numbers of differential genes of Δ*smpB* or Δ*ssrA* compared with wild type were analyzed through histogram (**A**,**B**) or Venn analysis (**C**,**D**) under LB medium (**A**,**C**) or M9 medium (**B**,**D**) conditions.

Under M9 culture conditions, the Δ*ssrA* and Δ*smpB* strains exhibited 434 (279 upregulated, 155 downregulated) and 338 (199 upregulated, 139 downregulated) differentially expressed genes (DEGs) compared to the wild type, respectively (Figure 4A). Under LB conditions, these numbers were 218 (187 upregulated, 31 downregulated) and 87 (35 upregulated, 52 downregulated) (Figure 4B). The total number of DEGs was significantly higher under M9, indicating greater reliance on tmRNA and SmpB in nutrient-deficient conditions (Figure 4A,B). Venn diagram analysis showed that 121 genes were co-regulated by Δ*smpB* and Δ*ssrA* under M9 (Figure 4D), while only 13 were co-regulated under LB (Figure 4C). These results suggest that SmpB and tmRNA cooperate more tightly under nutrient-deficient conditions (Figure 4A,B). The Δ*smpB* strain had 338 and 87 DEGs under M9 and LB conditions, respectively, with more pronounced changes in M9. This correlates with the decreased expression of the *smpB* gene in LB and increased expression in M9 (Figure 1D), indicating that SmpB is primarily activated under nutrient-deficient conditions to stimulate the trans-translation system and thus regulate downstream genes. We hypothesize that bacteria activate the trans-translation system under nutrient deficiency by regulating *smpB* expression, with tmRNA and SmpB cooperating in this function.

**Figure 5:**
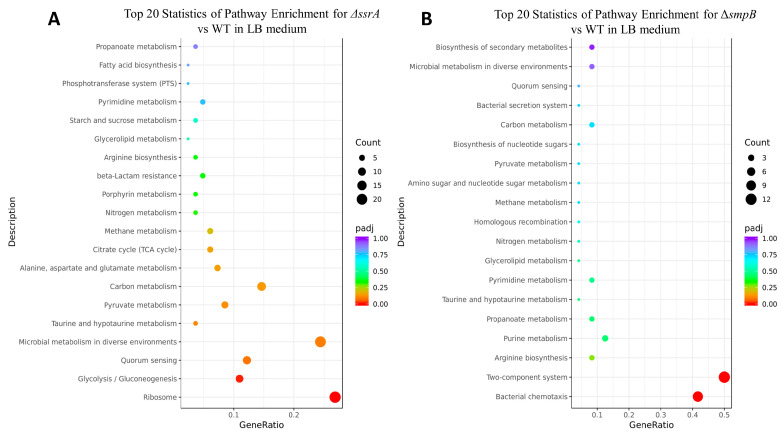

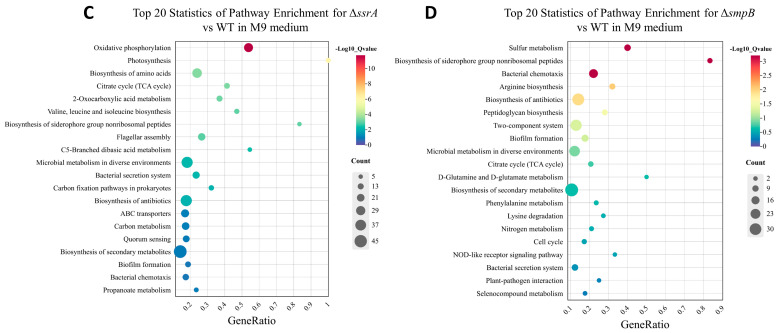
Functional enrichment analysis of DEGs based on the KEGG database. Top 20 statistics of pathway enrichment for Δ*ssrA* vs. WT (**A**,**C**) and Δ*smpB* vs. WT (**B**,**D**) in LB medium (**A**,**B**) and M9 medium (**C**,**D**).

**Figure 6:**
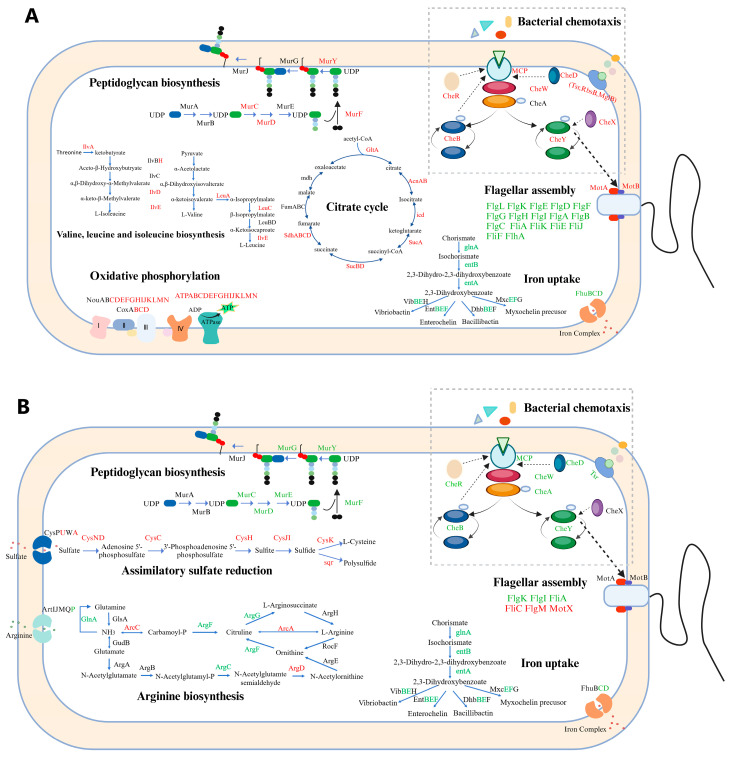
A model of the changes in cellular processes in Δ*ssrA* (**A**) and Δ*smpB* (**B**) as compared with the wild type based on the highly enriched pathways under M9 culture conditions. Red, green, and black marked genes indicate those with significant upregulation (FC > 2 and *p*-value < 0.05), significant downregulation (FC < 0.5 and *p*-value < 0.05), and no significant regulation (0.5 ≤ FC ≤ 2 or *p*-value ≥ 0.05), respectively.

In addition, we also noticed that, under both nutrient-deficient and nutrient-rich conditions, the Δ*ssrA* strain exhibited significantly more DEGs compared to the wild type than the Δ*smpB* strain (Figure 4A,B). This tendency was significant in the number of upregulated genes, indicating that tmRNA may also play an important role in gene expression regulation, alongside its collaboration with SmpB in trans-translation. KEGG pathway enrichment analysis revealed that, under LB conditions, the differentially expressed genes in the Δ*ssrA* strain were mainly enriched in ribosome biogenesis, microbial metabolism in diverse environments, and the tricarboxylic acid (TCA) cycle (Figure 5A, Table S2). Under M9 conditions, enrichment was observed in oxidative phosphorylation, amino acid biosynthesis, and the TCA cycle (Figure 5C, Table S3), suggesting increased demands for protein synthesis due to slow ribosome recycling from trans-translation defects, similar to the changes seen in *Bacillus subtilis* lacking *ssrA* [28]. In contrast, the Δ*smpB* strain showed that, under both LB (Figure 5B, Table S4) and M9 (Figure 5D, Table S5) conditions, the DEGs were primarily enriched in two-component systems and chemotaxis pathways, with additional enrichment in sulfur metabolism and the biosynthesis of siderophore groups and non-ribosomal peptides under M9 conditions. We also obtained models for the changes in the cellular processes in Δ*ssrA* and Δ*smpB* based on the highly enriched pathways under the M9 culture conditions (Figure 6A,B). These findings indicate that deletions of *ssrA* and *smpB* result in distinct gene changes, highlighting their different roles in gene regulation, influenced by the nutrient availability.

### 2.5. Cooperation of tmRNA and SmpB in Maintaining Iron Utilization in Pathogenic Bacteria

**Figure 7:**
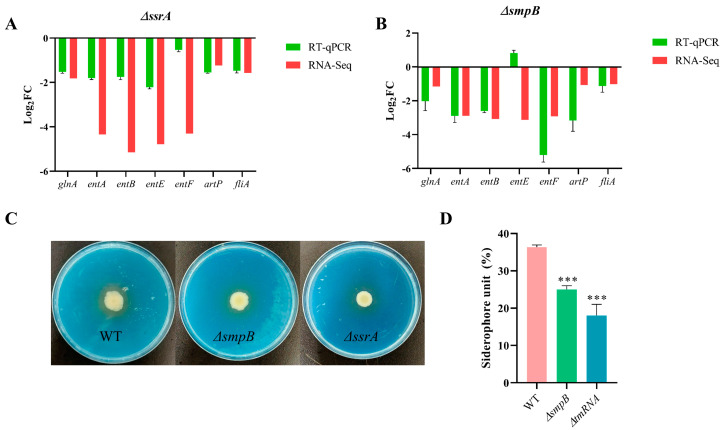
tmRNA and SmpB cooperatively regulate siderophore synthesis. RT-qPCR validation of genes involved in siderophore synthesis in Δ*ssrA* (**A**) and Δ*smpB* (**B**). Qualitative and quantitative analysis of siderophore formation. For qualitative analysis (**C**), 5 μL bacterial suspensions were cultured on CAS agar plates at 30 °C for 5 days. The yellow halo shows that siderophores produced by bacteria can strip the blue complex formed by cas and Fe^3+^ from the medium, and the wild type produces a darker yellow halo. For quantitative analysis (**D**), the bacteria were cultured in LB medium for 36 h, followed by centrifugation at 10,000 rpm for 10 min. Then, 100 μL supernatant was mixed with an equal volume of cas detection solution, and the absorption value at 630 nm was measured after standing in the dark for 1 h. Error bars represent standard deviations of triplicate experiments. Tukey’s post-test was used to assess statistical significance, with *** representing *p* < 0.005 in one-way ANOVA.

The KEGG pathway enrichment analysis revealed that the deletion of both *smpB* and *ssrA* significantly reduced the expression of genes associated with the siderophore and non-ribosomal peptide biosynthesis pathways under M9 conditions (Figure 5C,D). Additionally, we also observed that *smpB* and *ssrA* jointly regulated the expression of genes related to the ferric hydroxamate uptake (Fhu) system, specifically *fhuB*, *fhuC*, and *fhuD*, as well as the gene for the transmembrane transport protein tonB (Figure 6A,B). During inflammation, the host secretes antimicrobial proteins to sequester iron, zinc, and manganese ions, thereby limiting microbial growth. In response, pathogenic bacteria develop iron transporters and related systems to overcome iron availability challenges [30]. We first validated the expression levels of genes involved in iron transporter synthesis, including *glnA*, *entA*, *entB*, *entE*, and *entF*, in the Δ*ssrA* and Δ*smpB* strains using RT-qPCR. The results showed a significant decrease in expression, consistent with the transcriptomic sequencing data (Figure 7A,B). Further assessment of the siderophore synthesis capabilities was conducted using CAS agar plates, where the wild type produced a deeper yellow chelation zone compared to Δ*ssrA* and Δ*smpB* (Figure 7C). Quantitative analysis of the CAS detection liquid also indicated a significant reduction in iron transporter production in Δ*ssrA* and Δ*smpB* compared to the wild type (Figure 7D). These findings elucidate the cooperation of tmRNA and SmpB in the regulation of siderophore synthesis and indicate the potential molecular mechanism underlying the significant growth retardation observed in Δ*ssrA* and Δ*smpB* under treatment with 200 μM 2,2′-bipyridine (Figure 2C).

## 3. Discussion

mRNA synthesis can be interrupted by various events, including premature transcription termination, nuclease activity, and physical damage. When the ribosome reaches the 3′ end of a truncated mRNA, it becomes trapped in an incomplete translation complex [31]. The tmRNA-SmpB system specifically recognizes these stalled translation complexes and releases the blocked ribosomes, which is crucial in maintaining the cell’s protein synthesis capacity. Under adverse growth conditions, the RNase toxin components of toxin–antitoxin systems, such as RelE and MazF, can cleave most intracellular mRNA, resulting in the production of a large amount of truncated mRNA, which allows cells to conserve resources during periods of severe stress [32]. Under optimal growth conditions, these toxins are activated only in a small fraction of cells [33]. *E. coli* mutants lacking trans-translation activity show defects in recovering from toxin-induced stasis, indicating that trans-translation is important in resuming growth after prolonged severe nutritional stress [34]. The findings of this study further elucidate the molecular mechanisms by which the trans-translation system responds to severe nutritional stress. First, our detection of the expression levels of the *ssrA* and *smpB* genes indicates that the trans-translation system is activated under nutrient-deficient conditions through the responsive expression of *smpB*. This also helps to explain why strains with a single knockout of *smpB* are more vulnerable under the same nutritional stress conditions compared to strains with a single knockout of *ssrA*. Second, our transcriptomic analysis reveals that the total number of differentially expressed genes and co-regulated genes in the Δ*ssrA* and Δ*smpB* strains under M9 conditions is higher than that under LB conditions. The increase in co-regulated genes under nutrient-poor conditions can be considered evidence of the enhanced cooperation between tmRNA and SmpB, highlighting the importance of the trans-translation system in nutrient deficiency. In addition, we also found that tmRNA is stably expressed throughout the growth cycle of *A. veronii*, which is consistent with reports of stable tmRNA expression in *Staphylococcus aureus*, where tmRNA was used as a reference gene [35].

The results of this study indicate that tmRNA and SmpB collaborate and specialize in the response of pathogenic bacteria to stress and in carbon source utilization, with the transcriptomic analysis providing a series of molecular insights into these phenotypic observations. Here, we further validated the impact of tmRNA and SmpB on the iron uptake capabilities of *A. veronii*. Both the RT-qPCR and phenotypic experiments demonstrated the positive role of tmRNA and SmpB in the synthesis of siderophores. Additionally, when pathogenic bacteria are exposed to osmotic pressure from the environment, peptidoglycan plays a crucial role in protecting the bacteria from external and cytoplasmic stress while maintaining their cellular morphology [36]. Bacteria sense physical stimuli related to changes in external osmotic pressure and promptly regulate genes to sustain cellular viability [37]. Transmembrane signal transduction primarily relies on two-component systems (TCS) and membrane-bound chemical receptors, such as the components of chemotaxis systems [38]. The KEGG pathway enrichment analysis revealed that the deletion of *smpB* led to the significant downregulation of genes associated with the peptidoglycan synthesis pathway and chemotaxis systems, while the deletion of *ssrA* had the opposite effect on the same set of genes. This initially explains the significant growth inhibition observed in the Δ*smpB* strain under 0.5 M NaCl stress conditions, while the growth of the Δ*ssrA* strain was even slightly higher than that of the wild type (Figure 2D). Furthermore, we found that the deletion of *ssrA* led to a significant reduction in the expression of arginine transporter-related genes *artM*, *artQ*, and *artP*, while the deletion of *smpB* resulted in a notable decrease in the expression of *artP*. Although bacteria can synthesize L-arginine on their own, it is energetically more advantageous to uptake L-arginine from the environment at the expense of ATP [39]. The changes in arginine transporter-related genes in the Δ*ssrA* and Δ*smpB* strains explain the slower growth of these strains compared to the wild type when L-arginine was the sole carbon source (Figure 4C).

The trans-translation system is commonly understood to regulate gene expression by using the peptide tag encoded by tmRNA to facilitate the rapid degradation of rescue proteins. For example, in *C. crescentus*, tmRNA targets multiple proteins involved in DNA replication, recombination, and repair, with *tmRNA* deletion causing delays in DNA replication initiation [40]. In our previous study, GlnA was identified as a substrate of tmRNA [41], and, here, we found that *glnA* was significantly downregulated in the Δ*ssrA* and Δ*smpB* strains. Additionally, we observed the significant downregulation of tonB, a gene encoding a transmembrane iron transporter, in both strains. Twelve TonB-dependent receptors in *C. crescentus* have been identified as tmRNA substrates [40]. This raises the question of how tmRNA’s tagging activity primarily affects the protein levels while also influencing the transcription levels. One possible explanation is that tmRNA tags certain transcription factors, such as σ*^k^* [12], thereby regulating downstream expression. Alternatively, tmRNA may directly bind to target mRNAs as sRNA. Future studies should explore tmRNA’s regulatory mechanisms in specific genes from these two perspectives.

The collaboration and functional roles of tmRNA and SmpB have been extensively studied, but research on their independent pathways and activities is limited [20]. Our transcriptomic results indicate that the KEGG pathways involving tmRNA and SmpB are inconsistent, with only 13 co-regulated genes (4.4% of the total DEGs) under LB conditions, providing direct evidence for their independent functions. In this study, we found that tmRNA and SmpB regulate peptidoglycan synthesis and bacterial chemotaxis gene expression with opposing activities. Previous research showed that tmRNA deletion increases the GlcNAc content in the cell wall and upregulates peptidoglycan biosynthesis genes, enhancing the resistance to osmotic stress [27]. Conversely, the deletion of *smpB* increases the sensitivity to high sodium osmotic pressure. These phenotypic results align with the transcriptomic changes, indicating a functional division between tmRNA and SmpB in cell wall synthesis and the osmotic stress response. Flagellum synthesis is energy-intensive and fundamental to bacterial chemotaxis [42]. We observed the significant downregulation of flagellar genes in the Δ*ssrA* strain, likely due to reduced protein synthesis and energy utilization, consistent with findings in *B. subtilis* [28] and *Y. pseudotuberculosis* [15]. However, the upregulation of multiple chemotaxis genes in the Δ*ssrA* strain contradicts observations in *B. subtilis* [28] and is inconsistent with the downregulation of flagellar genes in *A. veronii*. Given the unclear distribution, function, and regulatory relationships between chemotaxis gene clusters and flagellar synthesis genes in *A. veronii*, tmRNA may serve as a key clue in elucidating the regulatory mechanisms of chemotactic activity in this organism.

Although the transcriptomic analysis in this study has provided key evidence for the cooperative and independent roles of tmRNA and SmpB in *A. veronii*, most of which were confirmed by consistent phenotypic observations, we still note that potential confounding factors may be present in the transcriptomic data. Therefore, aside from strict standards for data processing, we believe that the use of complementary technologies could strengthen the conclusions obtained in this study. For example, proteomic comparisons between the Δ*ssrA* and Δ*smpB* strains under different nutritional conditions would further validate the results of the transcriptomic analysis and provide a more comprehensive understanding of the cellular processes. A multimodal approach could also uncover post-translational modifications and regulatory mechanisms that transcriptomic data alone may not reveal.

## 4. Materials and Methods

### 4.1. Strains and Media

*Aeromonas veronii C4* and its derivatives were cultivated in Luria–Bertani (LB, Bio-tech, Shanghai, China) medium or M9 (Bio-tech, Shanghai, China) medium with shaking at 30 °C. Then, 50 μg/mL ampicillin (Amp, Sigma, Burlington, MA, USA) was supplemented for the regular cultivation of *A. veronii* strains. CAS medium was used to measure siderophore production [43]. Each 1 L of CAS medium contained 20% sucrose 10 mL, 10% acid-hydrolyzed casein 30 mL, 1 mmol/L CaCl_2_ 1 mL, 1 mmol/L MgSO_4_ 20 mL, agar 15 g, and phosphate buffer 50 mL. The CAS detection staining solution was slowly added at 60 °C. To prepare the CAS detection staining solution, solution A was prepared by dissolving 0.079 g CAS (Chrome Azurol S) in 50 mL deionized water and then adding 10 mL 1 mmol/L FeCl_3_ solution (which contained 12 mmol/L HCl). Solution B was separately prepared by dissolving 0.069 g of hexadecyltrimethylammonium bromide (HDTMA) in 40 mL deionized water. Solution A was gradually added to solution B along the wall of the beaker, and the mixture was stirred gently until it was homogeneous.

### 4.2. Quantitative Real-Time PCR (RT-qPCR) Analysis

Total RNA was extracted from the specified cultures using the Bacterial Total RNA Isolation Kit (Shengong, Shanghai, China), and genomic DNA remnants were eliminated with gDNA Wiper Mix (Vazyme, Nanjing, China). After assessing the concentration and purity of the RNA, 1 μg of RNA was used as a template for cDNA synthesis with HiScript II QRT SuperMix (Vazyme, Nanjing, China). The resulting cDNA was then diluted for RT-qPCR reactions conducted on an ABI Prism^®^ 7300 instrument (ABI, New York, NY, USA), with fluorescence detection performed using the ChamQ SYBR Color qPCR Master Mix (Vazyme, Nanjing, China). The primer sequences are provided in Supplementary Table S1. The *gapdH* gene was selected as a reference for data normalization, and all primers were designed using the Primer Premier 6 software (Table S1). The relative expression levels were quantified using the 2^−ΔΔCT^ method [44].

### 4.3. Phenotypic Determination Under Stress Conditions

For the measurement of the growth curves, the overnight cultures were collected at an OD_600_ of 1, diluted 1:100 in LB medium, and cultivated in a 96-well microplate at 30 °C. The optical densities of the cultures were recorded using a microplate reader (Synergy H1, BioTeK, Paramus, NJ, USA) at 600 nm at 1 h intervals until 24 h. For the starvation test, stationary-phase cultures grown in LB were washed three times with phosphate buffer solution. The culture was diluted to the same OD_600_ in phosphate buffer and incubated without shaking at 30 °C for different durations. A ten-fold serial dilution of the bacterial suspension was prepared, and 3 μL of each dilution was spotted onto LB agar plates. For low iron and high sodium salt stress, the culture was washed 3 times with PBS and then diluted to the same OD_600_ value. A ten-fold serial dilution of the bacterial suspension was prepared, and 3 μL of each dilution was spotted onto LB agar plates supplemented with different concentrations of 2,2’-bipyridine or sodium chloride. For the growth curves measured under the other stress conditions, as mentioned in the Supplementary Materials, the wild type and its derived strains with an OD_600_ of 1 were transferred to LB medium supplemented with the corresponding compounds and cultivated in a 96-well microplate at 30 °C. The optical densities of the cultures were recorded at 600 nm at 1 h intervals until 24 h. For the determination of the growth curves in different pH conditions, the LB medium was adjusted with hydrochloric acid or sodium hydroxide to the specified pH value, filtered, and then inoculated with bacteria.

### 4.4. Carbon Source Utilization Capacity Testing

A BIOLOG ECO microplate (Biolog, Hayward, CA, USA), which is a commercial 96-well plate pre-immobilized with 31 different types of carbon sources, was used to analyze the carbon source metabolic function of *A. veronii*. Overnight cultures were diluted with sterile water to an OD_600_ value of 0.01. The BIOLOG ECO microplate was preheated to 25 °C before use, and 150 μL diluted cultural liquid was added to each hole of the microplate. Sterile water was added as the control. The absorption value at 590 nm was read by a microplate reader at 0 h, 24 h, 48 h, 72 h, 96 h, 120 h, and 144 h at 30 °C. A bar chart was created according to the absorbance values recorded for each type of carbon source in relation to the corresponding time points. The experiment was repeated three times.

### 4.5. RNA Sequencing and Bioinformatics Analysis

The strains were incubated in LB or M9 medium with the same initial OD_600_ indicative of the stationary stage. The cells were collected and lysed, and total RNA was extracted by phenol–chloroform. RNA degradation and contamination was monitored on 1% agarose gels. RNA integrity was assessed using the RNA Nano 6000 Assay Kit of the Bioanalyzer 2100 system (Agilent Technologies, Santa Clara, CA, USA). The clustering of the index-coded samples was conducted on a cBot Cluster Generation System using the TruSeq PE Cluster Kit v3-cBot-HS (Illumina, San Diego, CA, USA), following the manufacturer’s instructions. After generating the clusters, the library preparations were sequenced on the Illumina Novaseq platform, producing 150 bp paired-end reads. The image data obtained from the high-throughput sequencer were converted into sequence data (reads) using CASAVA base calling. The criteria for the filtering of the raw data included removing reads with adapters, removing reads containing ‘N’ (indicating uncertain base information), and excluding low-quality reads (where more than 50% of the bases had a Qphred score of ≤20). The clean data were then assessed for Q20, Q30, and GC content calculations. All subsequent analyses were conducted on these high-quality clean data. For the genome alignment of the filtered sequencing reads, we used the software Bowtie2-v2.4.4, setting the mismatch parameter to 2 while keeping the other settings at their default values. The resulting sequence was mapped to the reference genome of *Aeromonas veronii* C4 (NCBI reference sequence: GCF_008693705.1). The RSEM-v1.3.1 and DESeq2-v1.20.0 software was used to calculate the gene expression levels to compare and analyze the gene expression differences among the samples. The threshold values for significant differences were *p* < 0.05 and |log2FC| ≥ 1. The significantly differentially expressed genes were analyzed for GO and KEGG functional enrichment.

### 4.6. Siderophore Production Assay

For qualitative analysis, after the overnight culture was washed twice with PBS, the bacterial suspension was diluted to the OD_600_ value, and 5 μL of liquid was cultured on the CAS agar plate at 30 °C for 5 days. Siderophore formation was preliminarily determined by the size and color of yellow chelating rings around bacterial colonies. For quantitative analysis, overnight cultures were transferred to new LB medium at the same OD_600_ value and incubated at 30 °C for 36 h with shaking. A 1 mL bacterial solution was centrifuged at 10,000 r/min for 10 min, and 100 μL supernatant was added into the 96-well microplate and mixed with the CAS detection solution in an equal volume. After incubation for 1 h at room temperature, the absorbance value of the mixture (As) was measured at the wavelength of 630 nm. The absorbance of the uninoculated medium mixed with CAS reagent was determined as the reference ratio (Ar). The percent siderophore unit (SU) was calculated according to the formula [(Ar − As)/Ar] × 100%.

## Data Availability

The transcriptome data obtained under M9 conditions have been submitted to the NCBI Sequence Read Archive (SRA) database, with the GEO accession number GSE120603 and the project ID PRJNA1202361. Additionally, the transcriptome data under LB culture conditions can be accessed via the NCBI with ID number PRJNA1203056.

## Supplementary Materials

The supporting information can be downloaded at: https://www.mdpi.com/article/10.3390/ijms26010409/s1.
