# Supplementary material for: Cooperative and Independent Functionality of tmRNA and SmpB in Aeromonas veronii: A Multifunctional Exploration Beyond Ribosome Rescue
Source: Int J Mol Sci. 2025 Jan 6;26(1):409. doi: 10.3390/ijms26010409 (PMC11722516; doi:10.3390/ijms26010409)
Supplement: Supplementary file 1 [file ijms-26-00409-s001.zip › ijms-3380852-supplementary materials.pdf]

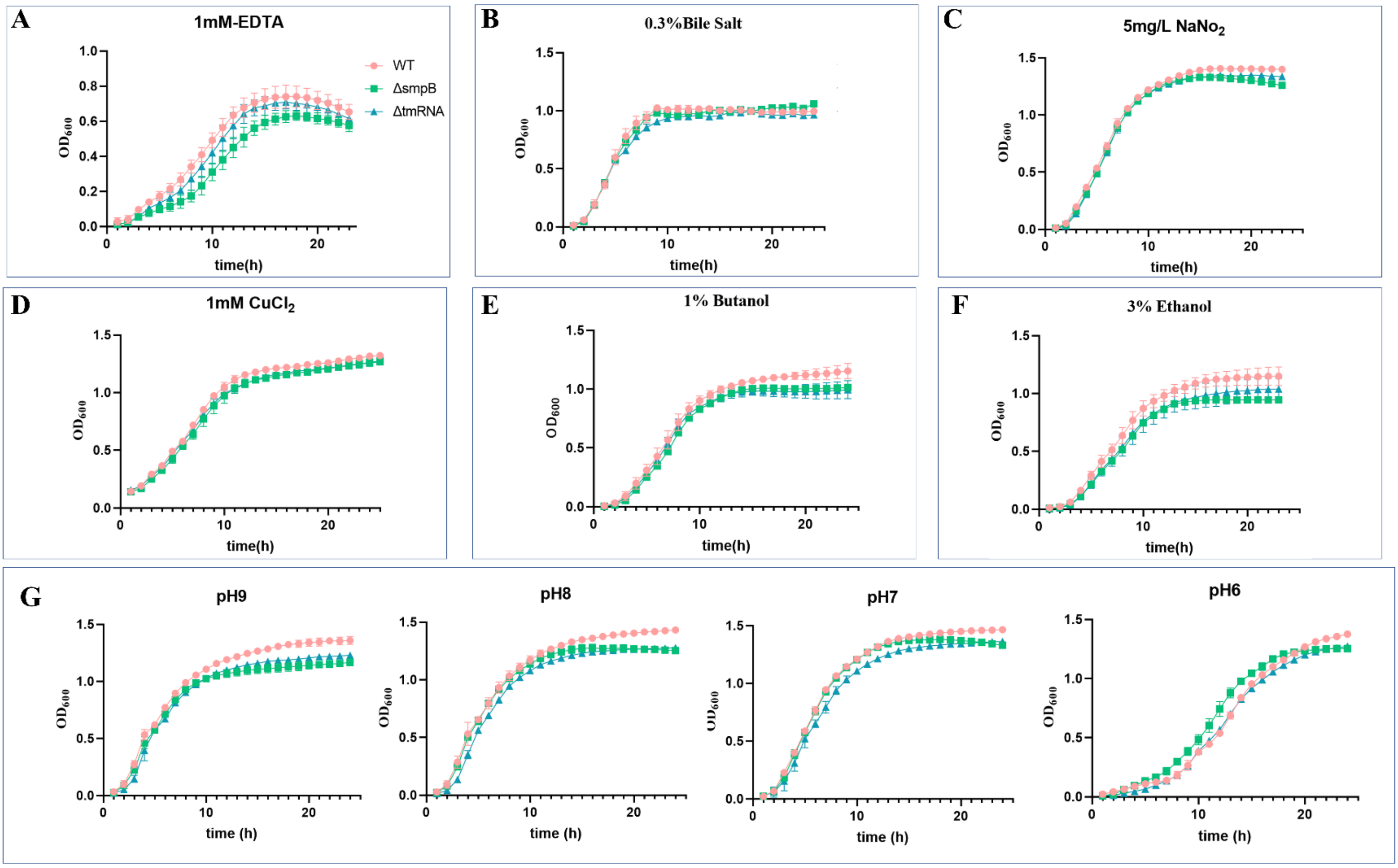

Supplementary Figure S1. Growth characteristics of WT and mutant strains in LB medium supplemented with 1mM EDTA (A), 0.3% bile salt (B), 5mg/L sodium nitrite (C), 1mM copper chloride (D), 1% butanol (E), and 3% ethanol (F). For growth characterization in different pH environments (G), LB medium was filtered after adjustment to the indicated pH and then inoculated with bacteria. Data were presented as mean± SD from three replicates.
